# Data on the pain of infiltration area in injection the anterior part of maxillary jaw

**DOI:** 10.1016/j.dib.2018.02.055

**Published:** 2018-02-28

**Authors:** Roohollah Sharifi, Hamid Reza Mozaffari, Hesameddin Nazari, Narges Ziaei, Arash Mohammadi, Seyed Mojtaba Amiri

**Affiliations:** a Department of Endodontics, School of Dentistry, Kermanshah University of Medical Sciences, Kermanshah, Iran; b Department of Oral Medicine, School of Dentistry, Kermanshah University of Medical Sciences, Kermanshah, Iran; c Department of Oromaxillofacial Surgery, School of Dentistry, Kermanshah University of Medical Sciences, Kermanshah, Iran; d Department of Periodontics, School of Dentistry, Kermanshah University of Medical Sciences, Kermanshah, Iran; e Faculty of Dentistry, Kermanshah University of Medical Sciences, Kermanshah, Iran; f Department of Biostatistic and Epidemiology, School of Health, Kermanshah University of Medical Sciences, Kermanshah, Iran

## Abstract

This article includes data from the research article entitled “The most painful site of maxillary anterior infiltrations“ (Sharifi et al., 2016) [1]. This article presents data of a study regarding pain from infiltration injection in the anterior region of maxilla in 3 locations in the buccal mucosa: the central and lateral incisors’ region as well as the canine region. Pain measurements were performed using the Visual Analogue Scale (VAS) at the time of needle insertion, as well as during injection of the anesthetic agent at 5, 30 and 55 s. The injectable agent included 1.8 ml lidocaine 2% with 1:80000 epinephrine.

**Specifications Table**

**Table:** 

Subject area	*Dentistry*
More specific subject area	*Pain from injection*
Type of data	*Table*
How data was acquired	*Visual Analog Scale (0–10)*
Data format	*Raw*
Experimental factors	*Single-blinded clinical trial was conducted on healthy volunteers.*
Experimental features	*Pain from infiltration injection in the buccal mucosa of the central and lateral maxillary incisors as well as the maxillary canine region at the time of needle insertion and during the injection of anesthetic agent was measured at 5, 30, 55 s.*
Data source location	*Kermanshah, Iran*
Data accessibility	*All of the data presented in this study are accessible within this article.*

**Value of the data**

• Data presented in this article was collected from a Single-blinded clinical trial.
• Measuring pain intensity in the anterior regions of maxilla during infiltration injection.
• Detecting the least painful site for a buccal infiltration injection in the anterior region of maxilla.
• Comparing pain intensity in the locations studied in this trial with future studies.
• Comparison of pain from injection in this study with pain from the injection locations in other studies.
• Documentation of patient's pain at any given time the researcher desired.

## 1 Data

The dataset of this article was obtained in Sharifi et al. [1]. Data includes gender information and pain intensity in different times and locations (Table 1).

**Table 1:** Pain from injection in each site in different locations and times.

id	Sex	Central				Lateral incisor				Canine			
		Insert	5^s^	30^s^	55^s^	Insert	5^s^	30^s^	55^s^	Insert	5^s^	30^s^	55^s^
1	Male	1.3	1.7	2.1	0.3	2.3	2	1	0	1	2.5	2	0
2	Male	1.5	3.1	3.2	1	1	2.2	1	0	1.5	3	1.5	0.5
3	Male	1.3	2.1	2	1.7	1.5	3	2	1	1.5	2.5	3	2
4	Female	2	3	1	1	1	1	0	0	1	2	0	0
5	Female	3	2	2	0	1	2	0	0	1	3	1	0
6	Female	2	3	3	3	4	5	2	2	3	4	3	3
7	Female	4	8	8	5.2	4	5	4	3	6	4	5	1
8	Female	0	3	1	0	2	4	1	0	2	2	0	0
9	Female	3.5	5.8	2	0	7	6	3	0	6	5	2	0
10	Female	2	5	0	0	0	2	0	0	2	0	0	0
11	Male	1	1.5	0	0	1.5	6	1.5	0	1.5	1	0	0
12	Male	1	0	0	0	0	0	0	0	0	1	0	0
13	Male	2	1	0	0	0	1	0	0	1	2	0	0
14	Female	4	5	2	0	3	6	4	2	5	3	2	1
15	Female	2	2	1	0	0	2	3	3	6	1	3	2
16	Male	2	5	1	1	5	7	6	3	3	3	1	5
17	Male	2	1	0	0	2	5	1	1	1	1	0	0
18	Male	2	8	4	1	0	4	4	4	6	7	4	2
19	Female	2	5	0	0	0	1	0	2	1	0	0	0
20	Female	4	5	6	6	1.8	2	2.2	2.5	0.5	1.5	2	2
21	Male	2	2	1	0	2	3	1	0	1	2	1	0
22	Male	5	1	2.5	2	4	3	2	1	6	5	4	2
23	Male	4	6	1	0	4.5	5	3	0	4	4.5	1	1
24	Male	2	1	1	0	1	2	1	0	3.5	3	1	2
25	Male	2	3	1	1	1	2	0	0	3	3	1	0
26	Male	2	0	2	2	2	0	2	2	0.5	2	1.5	3
27	Female	4	0	2	0	0	2	0	0	0	5	0	0
28	Female	3	1	2	1	2	1	0	1	3	2	1	0
29	Female	1	0	0	0	2	0	1	0	2	1	2	1
30	Female	1	0	0	0	3	2	1	0	1	1	0	0

## 2 Experimental design, materials and methods

In this cross-over, single blinded clinical trial; 30 volunteers were examined. Inclusion criteria: general health; existence of 6 intact anterior teeth with good response to vitality tests. Exclusion criteria: hypersensitivity to the injectable agent containing lidocaine and epinephrine; use of analgesics, anti-depressants, or anesthetic agents in 2 weeks prior to the trial; pain in the 6 anterior teeth in response to concussion or palpation; and history of any kind of surgery in the anterior region of maxilla. The method of this study was approved by the committee of ethics in research (IRCT2014091714333n20). Infiltration injection was performed for all of the volunteers in three turns with 2-week intervals in the region adjacent to apices of the maxillary central and lateral incisors as well as the maxillary canines. For the infiltration injection a cartridge of 1.8 ml lidocaine 2% with 1:80000 epinephrine (persocaine-E) was administered. Injection was done in the deepest region of the buccal vestibule adjacent to the apices of the teeth in the same conditions for all volunteers. Pain intensity was measured immediately after insertion of the needle, and at 5, 30 and 55 s after the beginning of the injection using a Visual Analogue Scale from 0 to 10.
